# Histamine Activates Human Eosinophils via H_2_R and H_4_R Predominantly in Atopic Dermatitis Patients

**DOI:** 10.3390/ijms231810294

**Published:** 2022-09-07

**Authors:** Leonie Beyer, Aylin Sara Kabatas, Susanne Mommert, Holger Stark, Thomas Werfel, Ralf Gutzmer, Katrin Schaper-Gerhardt

**Affiliations:** 1 Division of Immunodermatology and Allergy Research, Department of Dermatology and Allergy, Hannover Medical School, Carl-Neuberg-Straße 1, 30625 Hannover, Germany; 2 Institute of Pharmaceutical and Medicinal Chemistry, Heinrich Heine University Duesseldorf, Universitätsstraße 1, 40225 Duesseldorf, Germany; 3 Department of Dermatology, Johannes Wesling Medical Center, Ruhr University Bochum Campus Minden, 32429 Minden, Germany

**Keywords:** atopic dermatitis, eosinophils, histamine, IL-18 receptor, IL-18, transcriptome analysis

## Abstract

Atopic dermatitis (AD) is maintained by a variety of cells and inflammatory mediators, including eosinophils and histamine. We recently reported that eosinophils from AD patients highly express the H_4_R. However, its immunomodulatory function in eosinophils is still largely unexplored. In this study, transcriptome analysis of blood eosinophils from AD patients stimulated with histamine and the H_4_R agonist ST-1006 revealed several regulated genes (e.g., IL-18R, IL-1RL1, PDE4B, CXCR4) involved in inflammation. Subsequently, the impact of histamine on one of the strongly regulated genes, the IL-18 receptor (IL-18Rα), was investigated in detail. Stimulation with histamine induced the upregulation of IL-18Rα at mRNA and at the protein level in human eosinophils, which was more pronounced in cells from AD patients than in cells from healthy controls. IL-18 was upregulated via histamine as well. After pre-incubation with histamine and IFN-γ, subsequent stimulation with IL-18 resulted in an increased ECP mRNA expression. The activation of eosinophils by histamine, in combination with IFN-γ and IL-5, was also accompanied by an upregulation of CD69. Thus, our results indicate a crucial role of histamine in the upregulation of the IL-18/IL-18R axis and in the activation of human eosinophils from AD patients.

## 1. Introduction

Atopic dermatitis (AD) is a common chronic inflammatory skin disease affecting up to 20% of children and 3% of adults [1]. Relapsing eczematous skin lesions and intense pruritus impair the patients’ health-related quality of life and present substantial financial challenges for them and the health system [2,3]. The inflammatory process in acute lesions is maintained by a Th2-driven immune response with the concomitant production of IL-4, IL-5, and IL-13. Continuing skin irritations lead to dermal infiltration with eosinophils, inflammatory dendritic cells, and macrophages, which induce a shift towards a Th1-driven immune response and IFN-γ production in the chronic stage [4].

The number of eosinophils in the blood and skin has been positively correlated with disease activity and severity [5]. At the inflammatory sites, eosinophils get in contact with histamine, mainly released from activated mast cells and basophils, which contribute to strongly elevated levels of this biogenic amine in the skin of AD patients [6]. Histamine mediates its pleiotropic effects via four G-protein coupled receptors (H_1_R, H_2_R, H_3_R and H_4_R) and is thereby involved in mediating pruritus and inflammation. Due to this dual mode of action of the H_4_R, the development of H_4_R antagonists has lately emerged as a new promising treatment option for AD patients in clinical trials and therefore substantiated the importance of research on the role of the H_4_R, especially in inflammatory and allergic diseases [7,8].

Previous studies have demonstrated that stimulation of the H_4_R induces chemotaxis and partial activation of eosinophils, including calcium influx, shape changes, and upregulation of adhesion molecules [9,10,11,12]. Moreover, we recently reported that the H_4_R is highly expressed in eosinophils from AD patients compared to healthy controls and psoriasis patients and is upregulated via various Th2 cytokines [13]. In the present study, we focused on the immunomodulatory impact of histamine in eosinophils. We performed a transcriptome analysis in histamine- and selective H_4_R-stimulated and non-stimulated eosinophils from AD patients to identify potential target structures. Thereby, we were able to disclose several AD-associated genes regulated by histamine and the H_4_R. Based on these findings, we sought to further investigate the expression and regulation of one of the most regulated genes in eosinophils, IL-18R, in the context of AD in the present study.

## 2. Results

### 2.1. Transcriptome Analysis of Histamine-Stimulated Eosinophils from AD Patients

**Figure 1:**
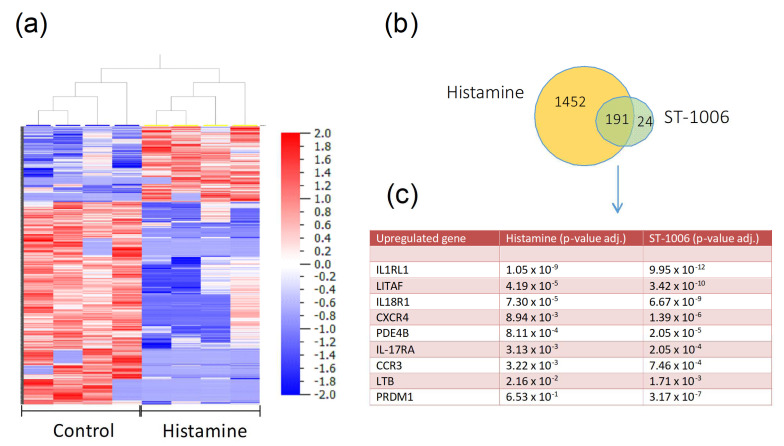
Histamine regulates several genes in eosinophils from AD patients. The heatmap shows the gene expression patterns of purified eosinophils from AD patients (*n* = 4) without treatment (control) and after stimulation with histamine (10 μM/mL) for 6 h (histamine) (**a**). It includes all significantly regulated genes. The legend shows the visualization of the log ratio (fold change). The genes that were upregulated are shown in red. Conversely, the blue areas indicate down-regulated genes. In total, 191 genes were significantly regulated by stimulation with both histamine and the selective H_4_R agonist ST-1006 (**b**). A selection of upregulated genes associated with AD is shown in a table (**c**).

To identify novel targets that are regulated by histamine and especially via H_4_R in AD patients, we performed a transcriptome analysis of purified human eosinophils from four AD patients, including a total of 58.721 genes (Supplementary Tables S1 and S2). In histamine-stimulated eosinophils, we detected a statistically significant regulation of 1643 genes that were either up- or down-regulated compared to the non-stimulated control (Figure 1a). Stimulation with the specific H_4_R agonist ST-1006 resulted in 215 statistically significant regulated genes, of which 191 were also significantly regulated by histamine (Figure 1b). Applying the Ingenuity Pathway Analysis Tool (IPA Tool; IngenuityHSystems, Redwood City, CA, USA; http://www.ingenuity.com, accessed on 21 January 2020), which is a software that transforms a list of genes into a set of relevant networks based on extensive records maintained in the Ingenuity Pathways Knowledge, we identified various networks of histamine signaling, which were primarily involved in inflammation and cell migration (Supplementary Figures S1 and S2). A selection of these genes is shown in Figure 1c. For further examinations, we focused on genes that were firstly regulated by both histamine and ST-1006, secondly highly expressed in eosinophils, and thirdly related to the pathogenesis of AD. As a result, we subsequently focused on the regulation of the IL-18Rα and its ligands for further examinations.

### 2.2. Histamine Induces Upregulation of IL-18Rα Expression via H_2_R and H_4_R in Human Eosinophils

**Figure 2:**
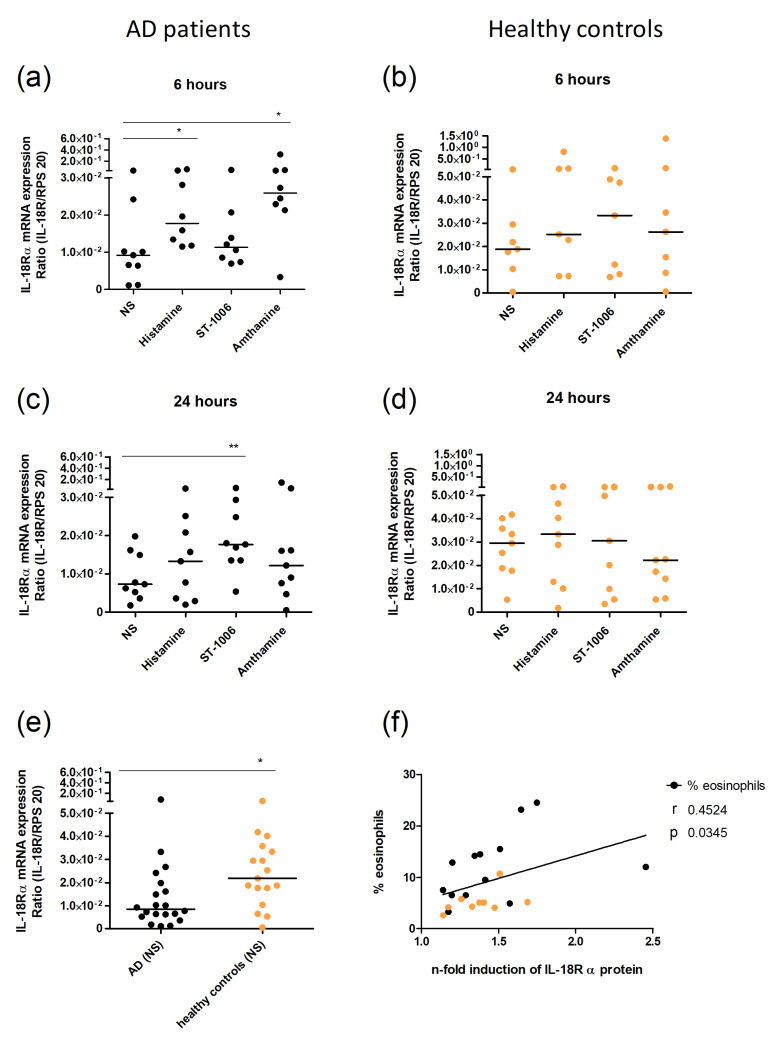
IL-18Rα expression in eosinophils compared between AD patients and healthy controls after stimulation with histamine, ST-1006, and amthamine. (**a**–**d**) Eosinophils from AD patients (black) and healthy controls (orange) were stimulated with histamine (10 µmol/L), the H_4_R agonist ST-1006 (10 µmol/L), and the H_2_R agonist amthamine (10 µmol/L) for 6 h (**a**,**b**) or 24 h (**c**,**d**) or were left unstimulated (NS). The expression level of IL-18Rα was measured at mRNA level by real-time PCR. (**e**) The constitutional IL-18Rα mRNA expression of non-stimulated (NS) purified eosinophils from AD-patients (black) and healthy controls (orange) is shown. (**f**) Eosinophils were stimulated with histamine (10 µmol/L) for 20 h and IL-18Rα expression was measured by flow cytometry. The extent of histamine-induced IL-18Rα expression (n-fold induction of IL-18Rα protein expression) was correlated with the number of eosinophils in the blood of each subject for statistical analysis, a Mann–Whitney test (**e**), a Spearman correlation (**f**), and a Wilcoxon signed rank test (**a**–**d**) were applied. * *p*-value < 0.05; ** *p*-value < 0.1. NS—not stimulated, AD—atopic dermatitis.

First, we confirmed the histamine-induced upregulation of the IL-18Rα at the mRNA level in eosinophils from AD patients by quantitative real-time PCR (Figure 2). Histamine as well as the H_2_R agonist amthamine increased the IL-18Rα mRNA expression after 6 h of incubation compared to the non-stimulated control only in eosinophils from AD patients (Figure 2a), but not in cells from healthy controls (Figure 2b). The H_4_R agonist ST-1006 induced a significant upregulation after 24 h of incubation only in cells from AD patients, while not in cells from healthy controls (Figure 2c,d). The constitutive IL-18Rα mRNA expression was significantly lower in eosinophils from AD patients when compared to cells from healthy controls (Figure 2e). Moreover, we found a positive correlation between the extent of histamine-induced IL-18Rα protein upregulation and the percentage of eosinophils in the blood (Figure 2f).

**Figure 3:**
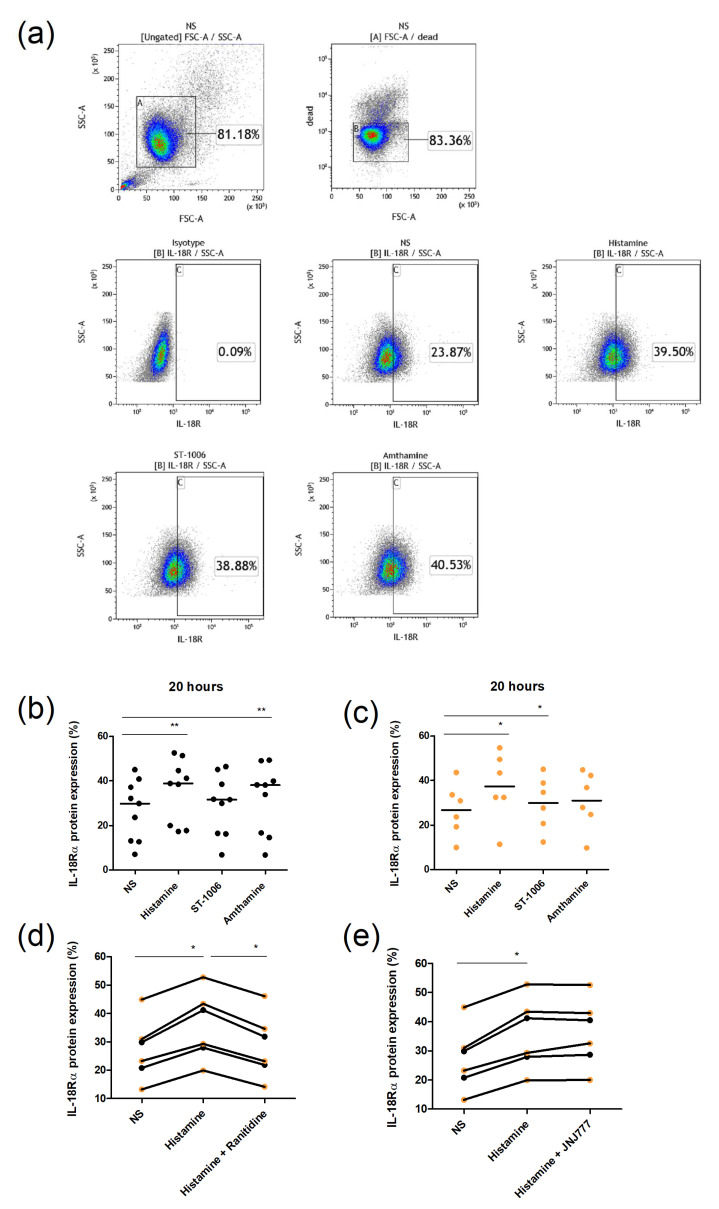
IL-18Rα protein expression upon stimulation with histamine, ST-1006, and amthamine. The expression level of the IL-18Rα on purified eosinophils was measured by flow cytometry. (**a**) Eosinophils from one representative donor were gated based on forward scatter (FSC) and side scatter (SSC) (A). Dead cells were labeled with the dye 7-AAD and excluded from the analysis by gate B. Gate C for IL-18Rα positive cells was set based on the isotype control. (**b**,**c**) Eosinophils from AD patients (black) and healthy controls (orange) were stimulated with histamine (10 µmol/L), the H_4_R agonist ST-1006 (10 µmol/L), or the H_2_R agonist amthamine (10 µmol/L) for 20 h or left unstimulated (NS). (**d**,**e**) For blocking experiments, the H_4_R antagonist JNJ7777120 (10 µmol/L) and the H_2_R antagonist ranitidine (10 µmol/L) were added 30 min before histamine. For statistical analysis, a Wilcoxon signed rank test was applied. * *p*-value < 0.05; ** *p*-value < 0.1. NS—not stimulated.

The surface expression of IL-18Rα was investigated by flow cytometry (Figure 3a). Consistent with the mRNA results, histamine and the H_2_R agonist amthamine, but not the H_4_R agonist ST-1006, induced an upregulation of IL-18Rα at the protein level in eosinophils from AD patients, while histamine and ST-1006 induced a slight upregulation in cells from healthy controls (Figure 3b,c). However, pre-incubation with the H_2_R antagonist ranitidine thoroughly inhibited the effect of histamine, whereas the H_4_R antagonist JNJ7777120 failed to block the upregulation of IL-18Rα surface expression (Figure 3d,e).

### 2.3. Histamine Induced Upregulation of IL-18 in Eosinophils from AD Patients

**Figure 4:**
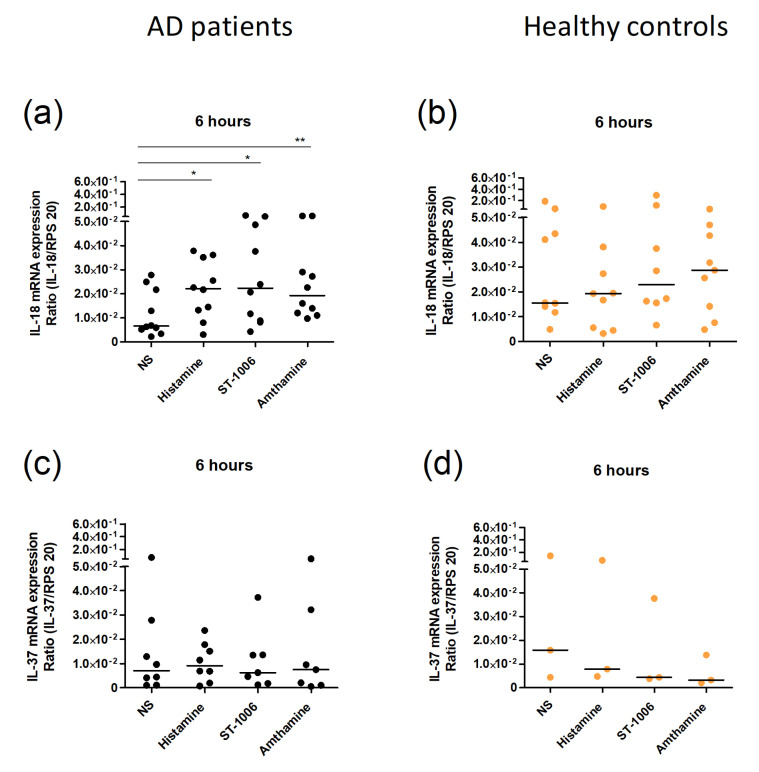
IL-18 and IL-37 mRNA expression after stimulation with histamine, ST-1006, and amthamine in eosinophils. (**a**–**d**) Purified eosinophils from AD patients (black) and healthy controls (orange) were stimulated with histamine (10 µmol/L), the H_4_R agonist ST-1006 (10 µmol/L), and the H_2_R agonist amthamine (10 µmol/L) for 6 h or were left unstimulated (NS). The expression level of IL-18 (**a**,**b**) and IL-37 (**c**,**d**) were measured at mRNA level by real-time PCR. For statistical analysis, a Wilcoxon signed rank test was applied. * *p*-value < 0.05; ** *p*-value < 0.1. NS—not stimulated.

The pro-inflammatory ligand of IL-18Rα, IL-18, was also found to be upregulated by histamine and ST-1006 in the transcriptome analysis. We confirmed this observation by q-PCR. Histamine, the selective stimulation of the H_2_R as well as of the H_4_R, increased the mRNA expression of IL-18 in AD patients (Figure 4a). Interestingly, again we could not observe an effect in healthy controls (Figure 4b). The other ligand of the IL-18Rα, the anti-inflammatory IL-37, was also regularly detectable at the mRNA level in eosinophils from AD patients and healthy controls. However, there was no significant difference in the IL-37 mRNA expression between histamine and non-stimulated cells (Figure 4c,d).

### 2.4. Histamine Enhances IFN-γ-Mediated Upregulation of IL-18Rα Expression via H_2_R and Reverses IL-5-Mediated Downregulation via H_4_R in Human Eosinophils

**Figure 5:**
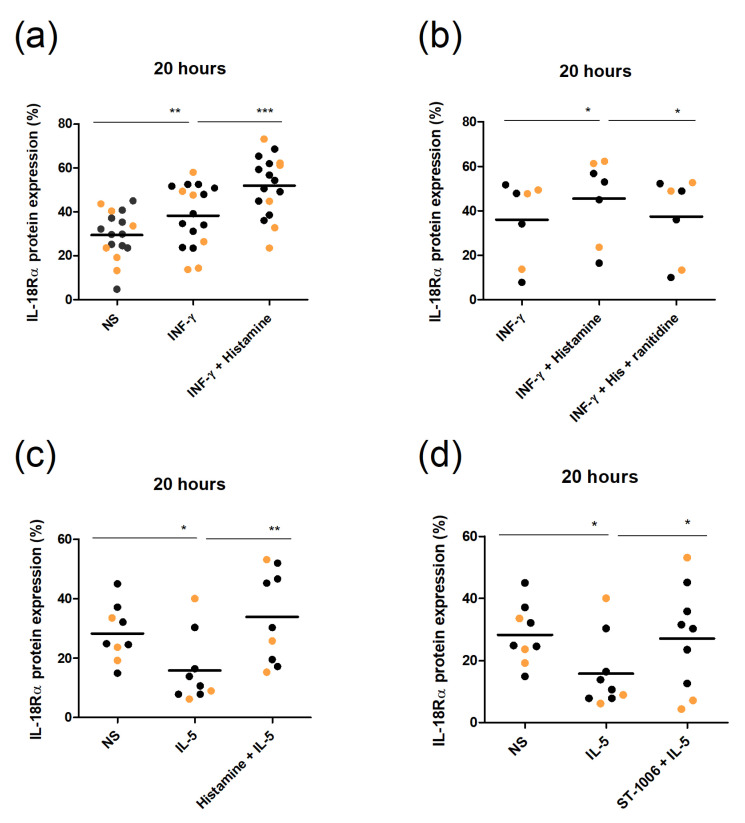
Histamine potentiates the IFN-γ-mediated IL-18Rα protein induction via H_2_R and abolishes the IL-5-mediated downregulation via H_4_R in eosinophils. The expression level of the IL-18Rα was measured by flow cytometry. (**a**,**b**) Purified eosinophils from AD patients (black) and healthy controls (orange) were stimulated with histamine (10 µmol/L), INF-γ (10 ng/mL), or the combination of both stimuli for 20 h. The H_2_R antagonist ranitidine (10 µmol/L) was added 30 min before histamine. (**c**,**d**) Purified eosinophils from AD patients (black) and healthy controls (orange) were stimulated with histamine (10 µmol/L), IL-5 (10 ng/mL), or the combination of IL-5 with histamine and H_4_R agonist ST-1006 (10 µmol/L), respectively, for 20 h. For statistical analysis, a Wilcoxon signed rank test was applied. * *p*-value < 0.05; ** *p*-value < 0.1; *** *p*-value < 0.01; NS—not stimulated.

In subsequent experiments, we investigated the impact of AD-associated cytokines and their interaction with histamine on the expression of IL-18Rα. The Th1 cytokine IFN-γ was observed to upregulate the IL-18Rα expression in eosinophils from AD patients (Figure 5a). The effect size was comparable with that of histamine (Figure 3b). Combined, histamine- and IFN-γ-stimulation showed an additive effect and strongly upregulated the IL-18Rα expression in eosinophils from AD patients and healthy controls, as shown here in one graph (Figure 5a). In healthy controls, these effects were less pronounced than in AD patients. Again, blocking of the H_2_R with ranitidine reversed the histamine-mediated upregulation (Figure 5b). The blockade of the H_4_R via JNJ7777120 had no effect (data not shown).

Next, we investigated the impact of the Th2 cytokines IL-4, IL-5, and IL-13 on IL-18Rα expression. We detected a significant downregulation of the IL-18Rα expression in eosinophils from AD patients after stimulation with IL-5 but not with IL-4 or IL-13 (data not shown). The combination of IL-5 with histamine reversed the downregulation of the IL-18Rα expression in eosinophils from AD patients and healthy controls (Figure 5c). Interestingly, IL-5 combined with the H_4_R agonist ST-1006 showed a similar effect (Figure 5d). 

### 2.5. Upregulation of the IL-18Rα Expression by Histamine Combined with IFN-γ in Human Eosinophils Is Functional

**Figure 6:**
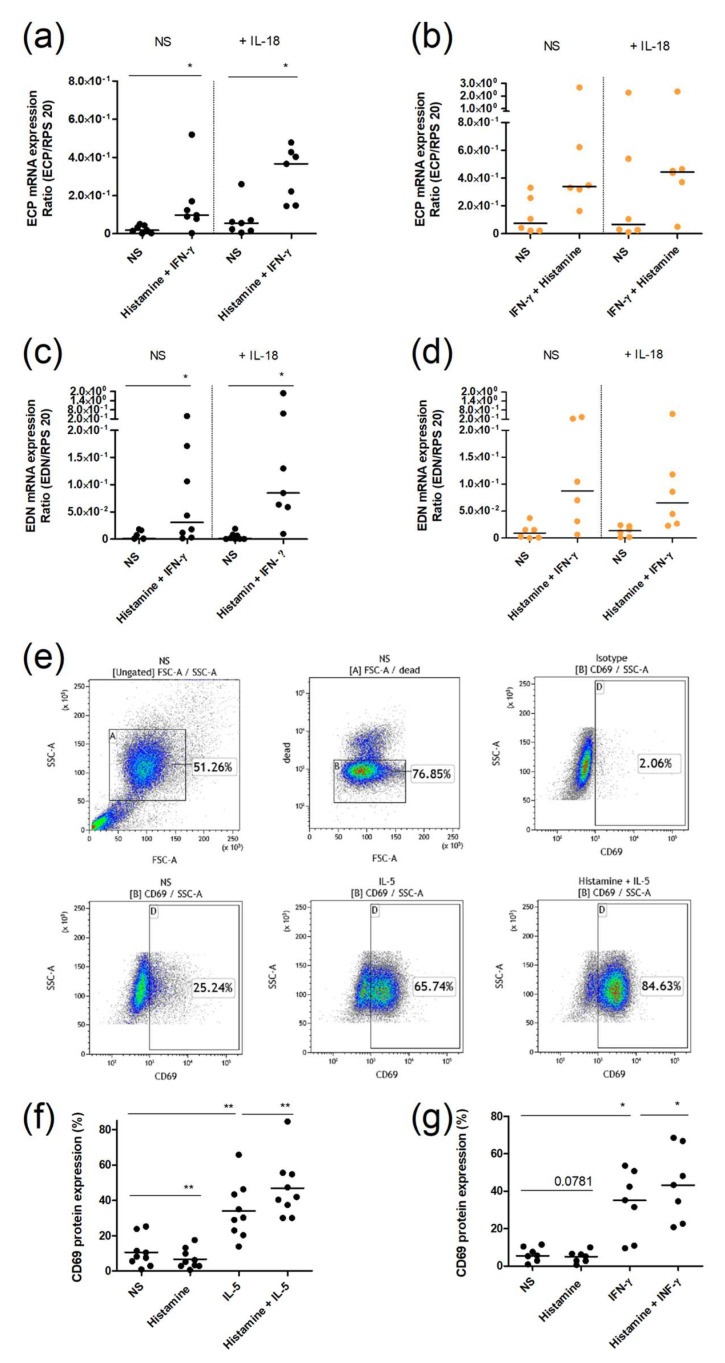
Histamine potentiates the activation of human eosinophils. (**a**–**d**) Purified eosinophils from AD patients (black) and healthy controls (orange) were stimulated in duplicate with histamine (10 µmol/L) combined with INF-γ (10 ng/mL) for 18 h or were left unstimulated. Cells were then washed with PBS and fresh culture media was added. Subsequently, one approach was stimulated with IL-18 (10 ng/mL) for 6 h, while the other approach was left unstimulated as control. The mRNA expression levels of ECP and EDN were measured by real-time PCR. (**e**–**g**) Purified eosinophils from AD patients (black) and healthy controls (orange) were stimulated with histamine (10 µmol/L), IL-5 (10 ng/mL), INF-γ (10 ng/mL), or in combinations of one cytokine each with histamine for 20 h. The expression level of CD69 was measured by flow cytometry. (**e**) Statistical summary graphs of dot blots showing one representative donor from stimulation (**f**). For statistical analysis, a Wilcoxon signed rank test was applied. * *p*-value < 0.05; ** *p*-value < 0.1; NS—not stimulated.

Since we have shown that stimulation of histamine receptors induces an increase in the IL-18R/IL-18 axis, we next wanted to check whether this upregulation also causes a functional effect in eosinophils. Thus, we decided to measure the expression of granule proteins from eosinophils. Therefore, we pre-incubated eosinophils with or without histamine combined with IFN-γ for 18 h to strongly upregulate the IL-18Rα. After washing, the cells were incubated with IL-18 for another 6 h or left unstimulated as control. The combination of histamine and IFN-γ increased ECP and EDN expression in both AD and healthy controls (Figure 6a–d). However, only in eosinophils from AD patients we observed a trend of an additional upregulation of ECP and EDN by additional incubation with IL-18 (Figure 6a,c).

In order to investigate a general activation by histamine, we also measured the surface molecule CD69, a known activation marker of eosinophils, by flow cytometry (Figure 6e). While histamine alone hardly showed an effect, the Th2 cytokine IL-5 (Figure 6f), as well as the Th1 cytokine IFN-γ (Figure 6g) significantly increased the protein expression of CD69. Interestingly, in combination with both cytokines, histamine displayed an additive effect regarding the activation of eosinophils (Figure 6e,g).

## 3. Discussion

In this study, we extend the previous understanding regarding the impact of histamine on eosinophilic granulocytes. During Th2-dominated inflammatory responses such as AD, eosinophils are recruited from the blood and bone marrow to the site of inflammation, where they have an important role in the development and maintenance of inflammatory diseases. Naidoo et al. identified eosinophils as key effector cells in the development of AD in the MC903 mouse model and pointed them toward a relevant therapeutic target for the clinical management of AD [14]. Moreover, Möbus et al. recently performed a blood mRNA sequencing study on 91 AD patients and segregated the population into two distinct clusters, with striking differences for transcripts involved in eosinophil signaling. Based on that, they divided AD patients into eosinophil high and eosinophil low endotypes [15]. Interestingly, one of the top central genes in the eosinophil-high AD-endotype group was H_4_R. Accordingly, we and others have previously shown that the H_4_R displays a particularly strong expression in eosinophils compared to other immunologically relevant cells [13].

By means of transcriptome analysis of eosinophils from AD patients, we have now identified several genes involved in the pathogenesis of AD, which are regulated by histamine and by the selective activation of the H_4_R. One of the top upregulated genes was IL-18Rα, which binds the pro-inflammatory cytokine IL-18.

IL-18 was initially described as IFN-γ-inducing factor in 1989 [16] but was later found to mediate pleiotropic functions depending on the surrounding cytokine environment [17]. Importantly, IL-18 is able to facilitate both Th1 and Th2 immune responses [18]. A number of allergic diseases, including AD, have been associated with elevated serum or tissue levels of IL-18 [19]. The impact of the overexpression of IL-18 in AD was studied by Konishi et al. in a transgenic mouse model with caspase-1-(KCASP1Tg) and IL-18-(KIL-18Tg) overexpressing keratinocytes. Here, IL-18 clearly contributed to the development of itchy, AD-like skin lesions in both transgenic mice lines, while the IL-18-deficient control exhibited none of the symptoms [20]. In line with this, IL-18 knockout mice were shown to have less pronounced skin lesions after being challenged with MC903 [21]. Due to the reported impact of IL-18 in the pathogenesis of AD, we selected IL-18R/IL-18 from our transcriptome analysis for further investigation.

We showed in our study that stimulation with histamine via H_2_R and H_4_R increases the IL-18Rα expression in eosinophils from AD patients at the mRNA as well as at the protein level. The simultaneous involvement of these two histamine receptors, activating different G protein-coupled signaling pathways, has already been shown in IL-27 downregulation of antigen-presenting cells [22]. These different molecular signaling pathways of the involved G protein-coupled receptors could also be a reason for the temporally different regulation of IL-18Rα expression. 

Under neutral cell culture conditions, the IL-18Rα upregulation was mainly mediated via the H_2_R. Although activation of the H_2_R is predominantly associated with an immunomodulatory role, we and others have already shown that the H_2_R is able to mediate pro-inflammatory effects as well. For example, stimulating M2 macrophages with an H_2_R agonist led to an increase in IL-4- and IL-10-induced CCL18 production [23]. Moreover, Kohka et al. reported that IFN-γ protein expression can be significantly increased on human PBMC by histamine via the H_2_R [24].

Conversely, we showed that the Th1-cytokine IFN-γ upregulated the IL-18Rα expression in eosinophils from AD patients, which has also been shown in other cell types, such as on human MoDC [25]. This IFN-γ-induced IL-18Rα upregulation in eosinophils in our study could be further amplified via stimulation with histamine, which was again mediated via the H_2_R.

Interestingly, we found that eosinophils obtained from AD patients have a significantly lower basal expression of the IL-18Rα as compared to healthy controls. A similar observation was made by Hou et al. showing decreased IL-18Rα expression in CCR3 positive blood cells from AD patients when compared to healthy controls [26]. Thus, we were questioning if Th2-associated cytokines may have an impact on IL-18Rα regulation. Indeed, we found that upon stimulation with the Th_2_-cytokine IL-5, but not with IL-4 and IL-13, the IL-18Rα expression was significantly decreased in eosinophils from AD patients. Taking into account that IL-5 levels are elevated in the serum of AD patients and also correlate with disease severity [27,28], this could be an explanation for the lower expression level of IL-18Rα on these cells.

However, in this study, we showed that stimulation with histamine and selective stimulation of the H_4_R were able to reverse the inhibitory effect of IL-5. Thus, during an inflammatory process, when eosinophils enter the tissue, we hypothesize that they come into contact with histamine, which has been reported to be strongly present in the inflamed skin of AD patients [29] and subsequently upregulates the IL-18Rα. In line with this hypothesis, the IL-18Rα expression in human keratinocytes from AD patients was shown to be significantly higher as compared to healthy donors, which can be related to the elevated histamine levels in inflamed skin [30]. Moreover, in the same study, the authors showed that keratinocytes functionally respond to IL-18 with upregulation of MHC II and production of the chemokine CXCL10/IP-10, which promotes a pro-inflammatory milieu.

We further checked the influence of histamine on the ligands of the IL-18R. We showed that eosinophils express IL-18 mRNA and that it is significantly upregulated by histamine in AD patients but not in healthy controls, maybe due to a higher baseline IL-18 mRNA expression in healthy controls. Interestingly, Venkateshaiah et al. have shown that IL-18 was able to transform IL-5-generated eosinophils into distinct inflammatory CD101^+^ and CD274^+^ expressing eosinophils [31]. Analyses of this subset revealed that IL-18-derived eosinophils become bigger in size and more granular. Thus, via increasing IL-18 and IL-18R expression in eosinophils, histamine might positively contribute to an autocrine mechanism resulting in a pathological phenotype. Moreover, considering that mast cells express the IL-18R [32,33] and that crosstalk between mast cells and eosinophils contributes substantially to allergic and non-allergic inflammation, it might be possible that histamine enhances this crosstalk via the IL-18/IL-18R axis. Moreover, it is tempting to speculate that an increase in IL-18 expression could result in an enhanced migration capacity to the side of inflammation, as has been shown for human MoDCs [25].

Just recently, it was reported that the anti-inflammatory opponent of IL-18, namely IL-37, is significantly decreased in the serum of AD patients [26]. Similar to IL-18, IL-37 binds to the IL-18Rα that subsequently heterodimerizes with the IL-1R8. However, although we were able to detect IL-37 mRNA in eosinophils regularly, histamine did not show any impact on the expression.

It has been shown that eosinophils increase the production of their granule proteins in response to IL-18 [31,34]. Upregulation of IL-18Rα accompanied with a local enhancement of the pro-inflammatory activity of IL-18 in the skin lesions might therefore cause activation and degranulation of eosinophils. In order to strengthen this hypothesis, we also measured the impact of histamine and IL-18 on the expression of cytotoxic granules and of CD69, a type II transmembrane protein, representing a very early activation antigen [35].

We showed that eosinophils from AD patients pre-incubated with IFN-γ and histamine response to the stimulation of IL-18 with elevated levels of EDN and ECP in comparison to eosinophils, which were not pre-incubated. In addition, we demonstrated that CD69 protein expression is increased after stimulation with histamine in combination with IFN-γ or IL-5. Since the degree of CD69 expression in eosinophils is supposed to be used as a biomarker for eosinophilic inflammation [33], histamine seems to clearly promote inflammation.

In conclusion, we demonstrated that histamine has an impact on various genes expressed by human eosinophils, which are involved in the pathogenesis of AD. In more detail, we showed that both the H_2_R and H_4_R, depending on the surrounding cytokine environment, are involved in the upregulation of the IL-18/IL18-Rα expression and further in the activation of eosinophils. However, since we did not verify the ligand-receptor-binding and the effects with respect to the H_4_R were only marginal, these observations must be interpreted with caution. Considering our results, the mutual interaction of IL-18/IL-18R, IFN-γ, IL-5, and histamine could therefore exacerbate the inflammatory process via eosinophils in an acute AD milieu dominated by Th2 cytokines or in a chronic disease stage dominated by Th1 cytokines. Moreover, our results underline the ability of histamine to facilitate the activation of eosinophils in AD, resulting in their further recruitment and increased degranulation. This suggests that H_2_R and H_4_R are potential therapeutic targets in AD and other inflammatory diseases where eosinophils are involved. While the use of H_2_R antagonists has not yet been successful in clinical trials in AD, H_4_R blockers have already shown anti-pruritic and anti-inflammatory effects in AD patients [7,8]. Interestingly, in a phase 2 study in patients with uncontrolled asthma, the H_4_R antagonist toreforant displayed significant improvements in a pre-specified subgroup, which displayed sputum eosinophils or blood eosinophils at baseline [36]. Due to the impact of histamine on eosinophil development and function, it is tempting to also speculate whether subgroups in AD patients with an increased number of eosinophils might especially benefit from H_4_R antagonists.

## 4. Materials and Methods

### 4.1. Subjects

In this study, a total of 35 patients with AD (mean age: 33.4 years) and 22 healthy subjects (mean age: 29.3 years) were included, whereby 4 AD patients were used for transcriptome analyses. AD patients did not receive systemic corticosteroid treatment for at least two weeks. Written consent was obtained from all subjects. The investigation of the role of histamine receptors in inflammatory skin diseases was approved by the local ethics committee of the Hannover Medical School (Vote No. 4253) and was conducted according to the Declaration of Helsinki Principles.

### 4.2. Isolation and Culture of Human Eosinophils

Venous blood was obtained from AD patients and healthy subjects. Eosinophils were isolated by Pancoll/Percoll-gradient centrifugation, erythrocyte lysis (lysis buffer: 155 mM NH_4_Cl; 10 mM KHCO_3_; 0.1 mM EDTA), and subsequent magnetic cell separation using anti-CD16 MicroBeads (Miltenyi Biotec, Bergisch Gladbach, Germany). Count and purity of the eosinophils were determined by cell staining with Kimura and light microscope examination (required purity ≥ 96%). Per well, 2–5 × 10^5^ eosinophils were cultivated in RPMI 1640 medium supplemented with 10% heat-inactivated fetal calf serum, 2 mM l-glutamine, 10,000 U/mL penicillin, and 10 mg/mL streptomycin (all Biochrom AG, Berlin, Germany) at 37 °C and 5% CO_2_. Cells were stimulated with histamine (ALK-Abelló, Hamburg, Germany), the selective H_2_R agonist amthamine (Tocris Bioscence, Wiesbaden-Nordenstedt, Germany), and the H_4_R agonist ST-1006 (Institute of Pharmaceutical and Medicinal Chemistry, Heinrich Heine University, Duesseldorf, Germany) as H_4_R agonist [37] for different time periods. The selective H_2_R antagonist ranitidine (Tocris Bioscence), and H_4_R antagonist JNJ7777120 (Sigma-Alderich, Darmstadt, Germany) were added 30 min prior to the stimulation. Furthermore, the cells were treated with IFN-γ, IL-4, IL-5 and IL-13 (all R&D Systems, Wiesbaden-Nordenstedt, Germany).

### 4.3. mRNA Isolation, Reverse Transcription, and Quantitative RT-PCR

After incubation, the harvested eosinophils were lysed, and mRNA was isolated with the RNeasy^®^ Mini Kit from Qiagen (Hilden, Germany) according to the manufacturer’s protocol. Reverse transcription for cDNA synthesis was carried out with the QuantiTect^®^ Reverse Transcription Kit from Qiagen. To perform quantitative real-time PCR, the Quantitect SYBR^®^ Green PCR Kit for RPS20 (QT00003290) was used with primers from Qiagen for IL-18 (QT00014560), IL-18R (QT00082922), IL-37 (QT00029904), ECP (QT00199731) and EDN (QT00203224). The amount of the target mRNA relative to the amount of the reference gene mRNA, ribosomal protein S20 (rps 20), in the same sample was calculated using the comparative Ct method also known as the ΔΔCt method provided by the Software LC 480 (Roche Molecular Biochemicals, Mannheim, Germany). The Ct values of both the calibrator and the samples of interest are normalized to the appropriate endogenous reference gene rps 20.

### 4.4. Library Generation

In total, 3 ng of total RNA were used for library preparation with the ‘SMARTer Stranded Total RNA-Seq Kit v2–Pico Input Mammalian’ (#634413; Takara/Clontech, Kyoto, Japan) according to conditions recommended in user manual #063017. Generated libraries were barcoded by dual indexing approach and were finally amplified by 12 cycles of PCR. Fragment length distribution of generated libraries was monitored using ‘Bioanalyzer High Sensitivity DNA Assay’ (5067-4626; Agilent Technologies, Santa Clara, CA, USA). Quantification of libraries was performed by use of the ‘Qubit^®^ dsDNA HS Assay Kit’ (Q32854; ThermoFisher Scientific, Waltham, MA, USA).

### 4.5. Sequencing Run

Equal molar amounts of twelve libraries in total were pooled for a common sequencing run. Accordingly, each analyzed library constitutes 8.3% of overall flowcell capacity. The library pool was denatured with NaOH and was finally diluted to 1.8 pM according to the *Denature and Dilute Libraries Guide* (Document # 15048776 v02; Illumina, San Diego, CA, USA). In total, 1.3 mL of denatured pool was loaded on an Illumina NextSeq 550 sequencer using a high output flowcell for paired-end reads (20024907; Illumina). Sequencing was performed with the following settings: Sequence reads 1 and 2 with 76 bases each; Index reads 1 and 2 with 8 bases each.

### 4.6. BCL to FASTQ Conversion

BCL files were converted to FASTQ files using bcl2fastq Conversion Software version v2.20.0.422 (Illumina).

### 4.7. Raw Data Processing and Quality Control

Raw data processing was conducted by use of nfcore/rnaseq (version 1.3), which is a bioinformatics best-practice analysis pipeline used for RNA sequencing data at the National Genomics Infrastructure at SciLifeLab, Stockholm, Sweden. The pipeline uses Nextflow, a bioinformatics workflow tool. It pre-processes raw data from FastQ inputs, aligns the reads, and performs extensive quality control on the results. The genome reference and annotation data were taken from GENCODE.org (Homo sapiens; GRCh38.p12; release 29).

### 4.8. Normalization and Differential Expression Analysis

Normalization and differential expression analysis was performed with DESeq2 (Galaxy Tool Version 2.11.40.2) with default settings except for “Output normalized counts table”, which was set to “Yes”.

The treatment was selected as primary factor, whereas the donor was used as secondary factor in DESeq2 analyses (paired design).

### 4.9. Flow Cytometric Analysis

Cultured eosinophils were harvested after 20 h of incubation since experience has shown that we detect a robust effect in protein expression of histamine receptor stimulation after approximately 18–24 h. Subsequently, we stained with the PE Mouse Anti-Human CD218a (IL-18Rα) antibody (BD Biosciences, San Jose, CA, USA) and the FITC Mouse Anti-Human CD69 (Biolegend, San Diego, CA, USA). In order to exclude unspecific binding, cells were also stained with the PE Mouse IgG_1_ κ isotype (IL-18Rα) or the FITC Mouse IgG_1_ κ isotype (CD69) as control (BD Biosciences). Surface expression of the IL-18Rα in the resuspended eosinophils was determined by means of flow cytometry (FACSCanto II System, BD Biosciences) and analyzed using the Kaluza software (Beckman Coulter, Brea, CA, USA).

### 4.10. Statistical Analysis

Statistical analysis was performed using GraphPad Prism 5.0 (San Diego, CA, USA). First, measured data were checked for normal Gaussian distribution. Secondly, not normally distributed samples from two groups were analyzed using a Wilcoxon matched-pairs test. For normally distributed samples, a paired *t*-test was applied. Unpaired samples from two groups were evaluated using a Mann–Whitney test. For all calculations, a 95% confidence range was defined. The level of significance was graphically indicated as follows: * *p*-value < 0.05; ** *p*-value < 0.01; *** *p*-value < 0.001.

## Data Availability

The data that support the findings of this study are available from the corresponding author upon reasonable request. RNAseq database is provided in the Supplementary Materials.

## Supplementary Materials

The following supporting information can be downloaded at: https://www.mdpi.com/article/10.3390/ijms231810294/s1.
