# Supplementary figures and images for: Histamine Activates Human Eosinophils via H2R and H4R Predominantly in Atopic Dermatitis Patients
Source: Int J Mol Sci. 2022 Sep 7;23(18):10294. doi: 10.3390/ijms231810294 (PMC9499661; doi:10.3390/ijms231810294)

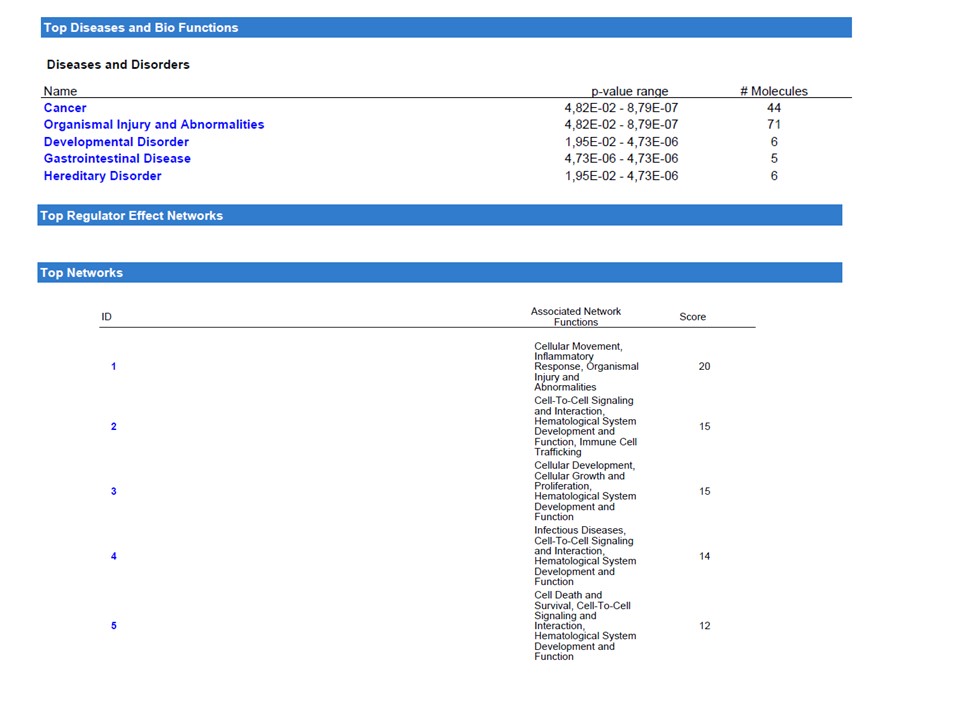

Supplement: Supplementary file 1 [file ijms-23-10294-s001.zip › Supplementary Figure S1. IPA Analyses His.jpg]

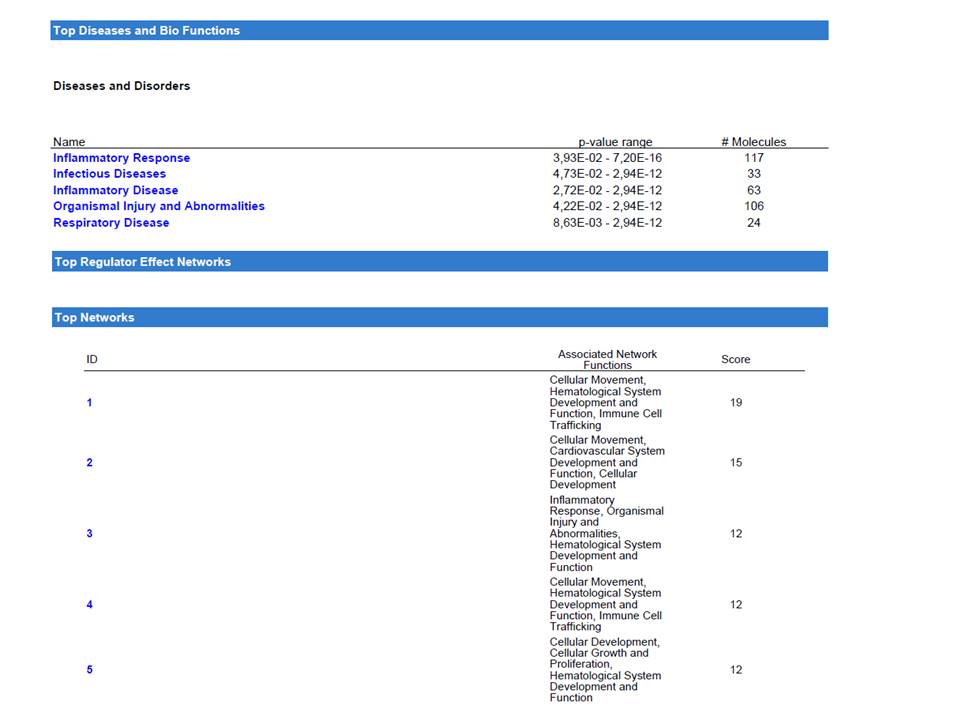

Supplement: Supplementary file 1 [file ijms-23-10294-s001.zip › Supplementary Figure S2. IPA Analyses ST1006.jpg]
